# A novel intelligent agent-based framework for appropriate stream selection from perceptive of career counseling

**DOI:** 10.7717/peerj-cs.1256

**Published:** 2023-02-22

**Authors:** Abdulrahman Abdullah Alghamdi

**Affiliations:** Computer Science Department, College of Computing and Information Technology, Shaqra University, Shaqra, Riyadh, Saudi Arabia

**Keywords:** Computer science, Intelligent systems, Career guidance, Learning, Information technology, Agent-based system, Stream selection

## Abstract

Picking a career stream profoundly influences people’s abilities in different ways. Nowadays, choosing the correct career stream in advanced education is troublesome, as the number of people wanting to be in specific specializations is growing. Therefore, it is essential to be able to select the appropriate career path. This article proposes a system that can suggest streams in advanced education schools. This system is influenced by agent-based stream proposal systems (ASPS). The proposed system aims to make picking out the correct stream to study at school simpler for an individual. Different streams are evaluated based on seven pre-characterized models. In our system, three unique sorts of tests, learning styles, and coaching were coordinated in a way that caused the system to recognize an individual’s interests and limits to an area of study. A sample of 238 participants was recruited for our questionnaire on accessibility, user-friendliness, accuracy, and satisfaction with the system. The incorporation of learning styles and coaching proved helpful in the study. The reliability and validity were proven in addition to incorporating a thinking-aloud protocol and immediate evaluation in the pre-, during and post-tests. To a large extent, respondents were satisfied with the model, as was revealed in the Likert scale response frequencies and percentages. The proposed system can be applied to undergraduates choosing engineering, medicine, arts and science degrees

## Introduction

Creating a computer program mimics a biological brain’s behaviour is the field of artificial intelligence. A program running on a computing device operates electronically instead of by triggering biological neurons (Walter, 1997). Artificial intelligence (AI) is the engineering discipline of creating intelligent computer systems (John, 2007). Expert systems (ES) are a good example of AI systems. Any machine with artificial intelligence can be referred to as an intelligent system. In today’s society, the use of intelligent systems is increasing in various areas, such as medical care, factory automation, visual inspections, education, entertainment, and optical character recognition (Abisoye et al., 2015).

Learning can be characterized as the exercises embraced to obtain or develop information, the principles dictated by general society and gained through explicit instructions based on information and abilities. Picking a correct learning stream is perhaps a critical decision made in everyday life. Stream selection can be supported for individuals with a reasonable degree (Razak et al., 2014). The principle intent of stream selection administration is to provide people with attributes and fields of knowledge towards the most suitable streams matched with their qualities and areas of interest (Jeon & Lee, 2015).

The term “career” has become an area of focus for the attention of experts. Stream selection is characterized by various cycles, strategies, or administration intended to help a person understand and pursue information on favourable circumstances in work, instruction, and relaxation and to build up the dynamic abilities to make and deal with their career advancement. In our work, stream selection corresponds to administration practices proposed to help participants to choose streams at school.

Choosing a major is not always easy for undergraduates, primarily because the selection should be based on a few characteristics and made at a very young age. This major decision affects the academic and professional lives of interested people and the efficacy of schools. Many educational and societal problems may be traced to poor instruction or expert advice, such as school failure, dropout, lack of skills, coordination challenges, and unemployed (Razak et al., 2014).

According to the likes and future yearnings, tutoring and building up students is a challenge, especially when it is dependent on assorted monetary and social foundations. Proposal systems are generally used to direct clients to discover the items or processes to accommodate their inclinations (Jeon & Lee, 2015; Zaharim et al., 2010) better. No such dynamic criteria system exists in the current advanced degree system. Because of a lack of help in career guidance administration and improvement support, a student’s career change after a college degree is not smooth (Talib & Aun, 2009). Many students pick their careers without appropriate advice and planning from experts or organization administration (D’Mello, 2016). This may cause a gap between scholarly accomplishments and students’ character, interests, and skills.

Let us assume a student aims to study for a Master of Business Administration (MBA). We can imagine that they are keen on specialized learning. Alternatively, we can imagine their interests are linked to gadgets but not extra-curricular activities. Additionally, some students would like to join higher studies following graduation, while others might want to join the industry. The second category may be less worried about research-oriented courses or courses that do not extend to employment opportunities in the industry. On the off chance that an educator does not know about their student’s interests, these issues can lead to clashes and non-participation of the students for a part of the exercises/courses. Therefore, it is essential to discover their interests through, for example, initial coaching.

Systems (Singh et al., 2018; Razak et al., 2014) have several disadvantages. For example, career guides are not consistently accessible according to a person’s abilities and preferences. In addition, guidance and direction may be restricted to only a few establishments resulting in not every student having access to advising services. Advisors and career guidance sites charge colossal fees for guidance that is not moderated for large social gatherings. Furthermore, many websites have unfriendly graphical user interfaces (GUI), and clients think they are hard to use. Hence, an improved, intelligent stream selection system is needed to provide successful career guidance.

ESGuiscar-web was developed as a web-based student career guidance system for students of vocational high schools (Katore, Ratnaparkhi & Umale, 2015). The ESGuiscar-web application supports or enhances career advice from career experts at schools and for parents. According to Herstatt & Kalogerakis (2006), this web-based application aims to assist students in obtaining career advice to determine which career options best fit their interests and potential. One of the benefits of ESGuiscar-web is that students can get independent career advice and see how their claims include the world of work, higher education, or starting a business. ESGuiscar-web is not a device that can fully guarantee the accuracy of guidance services. On the other hand, this scheme serves as a tool for students to pursue their passions (Supriyanto et al., 2019; Lent, Brown & Hackett, 1994). According to Haristiani & Firmansyah (2016), students need to carry the findings of their career guidance to school and consult with their parents and career guidance teachers. Aji et al. (2018) proposed a machine learning data mining technique that recommended that students continue their careers in given fields of research. They used datasets to finish their study. Gysbers & Henderson (2001) suggested a brilliant community level model for the resilience planning with the help of suitable agent selection method. An agent-based simulation framework was presented by Shahed, Rabby & Sarker (2020) which work using NetLogo, which was used for K12 Science Education aspirants. The agent based simulation was effective during the Covid19 pandemic outbreak. Aghababaei & Koliou (2022) presented an exciting model for the prediction of the effective countermeasures during this pandemic. This shows the effectiveness and efficiency of the agent based simulation techniques.

To improve the career suggestion system, two different algorithms were used. 60% accuracy was achieved using a naive Bayes classifier and 64% precision with k-NN, which is far from ideal. Kinner & Whitaker (2022) studied the influence of expert systems on student advice on educational counselling, educational assessment, aspects of academic career, and employment assistance. Expert education systems positively impact students’ learning progress, specialization in education and training, student achievement, all-around performance, and self-assessment. In this study, we present certain conventional stream selection strategies for the students. There are a couple of system which might not be accurate and the study further proposes a novel method for the same.

## Motivation of the proposed methodology

Issues with conventional stream selection strategies are as follows:

- Various strategies have been proposed to improve the execution of stream selection systems, yet they are not very accurate.
- AI-based methods have previously been used to improve career guidance systems’ precision.
- Techniques based on the systems were proposed for the stream selection system. Mistakes were assessed. The strategy was more proficient and vigorous, yet, it brought poor accuracy.

## Proposed methodology

**Figure 1:**
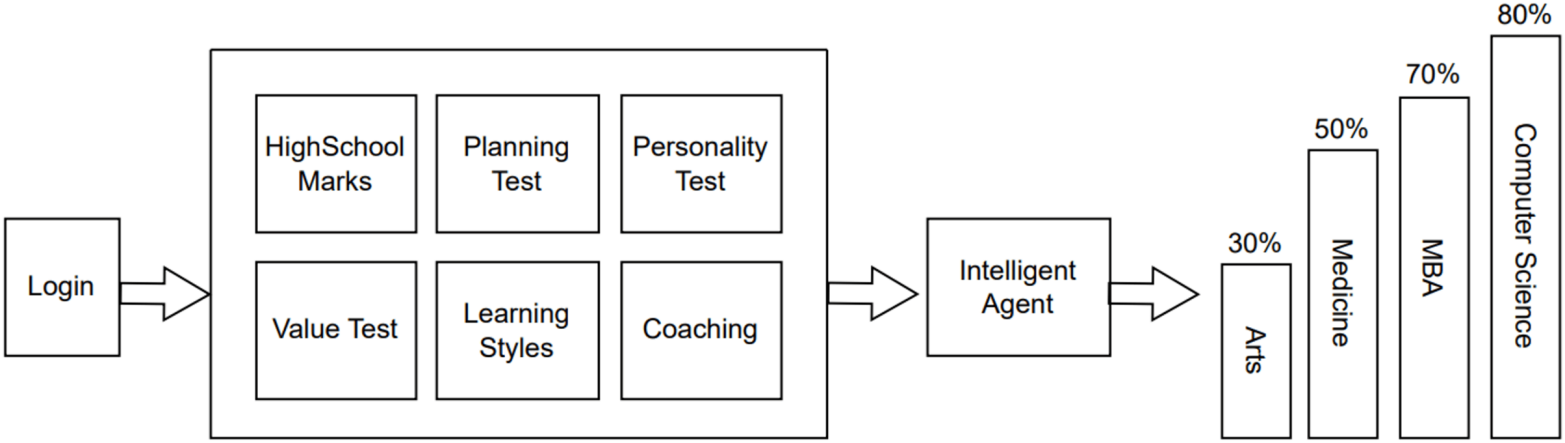
Workflow.

The proposed agent-based profession direction framework comprises three stages: (1) user management, (2) starting stream suggestion and (3) stream idea. Clients (*e.g*., students and executives) are supervised in the first stage. In the second stage, different streams are evaluated depending on seven pre-characterized measures discussed in “Conclusion and Future Perspectives”. In the third stage, the specific stream is suggested to the client in view of the outcomes acquired from the previous step. Specialists perform all these activities. This framework was planned to use specialist-based strategies, which were coherently applied to yield the proposed system. The general design of the proposed approach appears in Fig. 1.

### Agent-based systems

**Figure 2:**
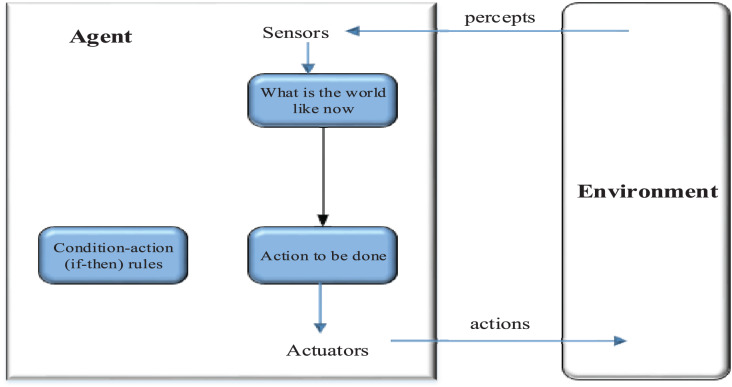
Environment interaction.

An agent is a self-governing computer-based framework, adaptable in nature, that can obtain information from its environmental context (Kinner & Whitaker, 2022). There are several benefits to making use of specialists. For instance, they can give information to do a particular mission and team up with one another, which is helpful when creating secure frameworks. A specialist can address a client and finish undertakings for this client. In addition, specialist frameworks are extendible. Another specialist can be added to a current framework to address another client, for example, an organization observing specialist, without changing the whole framework. The architecture of a specialist-based framework appears in Fig. 2. The selection of an agent is done based on the inputs and conditions. The actuators are defined to identify the actions that will interact with the environment. The percept from the results is identified and the final agent is chosen based on the results received in this case.

### Stream suggestion assessment

Interaction with the proposed system begins with the login stage. At this stage, the client is checked for legitimacy. Once the client is found eligible for the system usage, he is redirected to the access dashboard. There are several factors based on which the stream suggestion will take place. These factors are identified for the generic cohort of the students. After successful authentication, the stream idea evaluation is completed based on six standards:

- Marks obtained at high school;
- Planning test;
- Personality test;
- Value test;
- Learning style;
- Coaching.

### Marks obtained from high school

Subjects like physical sciences, biology, chemistry, studies of the planet, mathematics, English language, and computing are essential for secondary school instruction by the proposed system. The system will separate the subjects based on streams like medicine, engineering science, humanities, and software engineering. Subject classifications are one of the contributions of the astute agent.

### Personality tests

Isabel Briggs Myers and her mother, Kathryn Briggs, are devoted to studying the concepts of Carl Jung, a Swiss psychiatrist (Kersting et al., 2021). People prefer to rely on variables such as parental advice and emulation when choosing a job path rather than pursuing their own interests. While many individuals train hard in various professions merely because they think they are “cool,” many others have a genuine enthusiasm for the field they are working in. Every personality has strengths and weaknesses based on innate tendencies (Sakthiyavathi & Palanivel, 2009). One of the primary uses of the Myers-Briggs type indicator is for identifying potential career paths for people with different personalities. Personality type assessment may be used to discover a job that matches your personality type (Myers, 1998).

**Figure 3:**
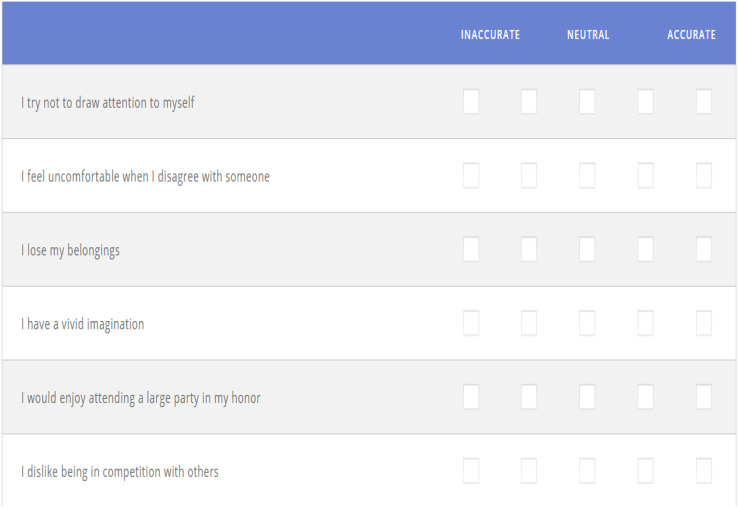
Survey.

Personality tests are used to analyze the character of the participants. These character tests are based on Myers and Briggs (Dunning, 2010). They provide clear and precise scores so that the participants can precisely know the character they fit the best. In this test, there are 30 kinds of affirmations. To take the test, answers ought to be checked based on how well every declaration portrays the participant’s character. They pick responses based on how they are, not how they might want to be. Figure 3 represents some example affirmations available in the character test.

### Value test-Holland code career test

The RIASEC theory of Holland (1997) was developed to solve the vocational problems of U.S. citizens of the mid-twentieth century. However, in many ways, principles of individualism, autonomous decision-making, and the structure of the immediate family are not the rule (Farr & Shatkin, 2009). As a result, others have questioned the contribution of Holland’s theory and its applicability in the global economy to U.S. people and the workers of the 21st century (Myers, 1962). The view of Holland is based on four basic assumptions, which characterize the development of occupational interests. It is assumed that individuals can be classified as realism (R), research (I), artistic (A), social (S), enterprise (E) and conventional (C). The second assumption is that societies (*e.g*., workplaces) often fall into six different categories. The third theory is that people prefer to select settings that match their personalities. The fourth assumption stresses the importance of the unity of one’s personality with their environment. It states that behaviour is dictated by the match between the personality and environment of a person (Stead & Watson, 1998).

**Figure 4:**
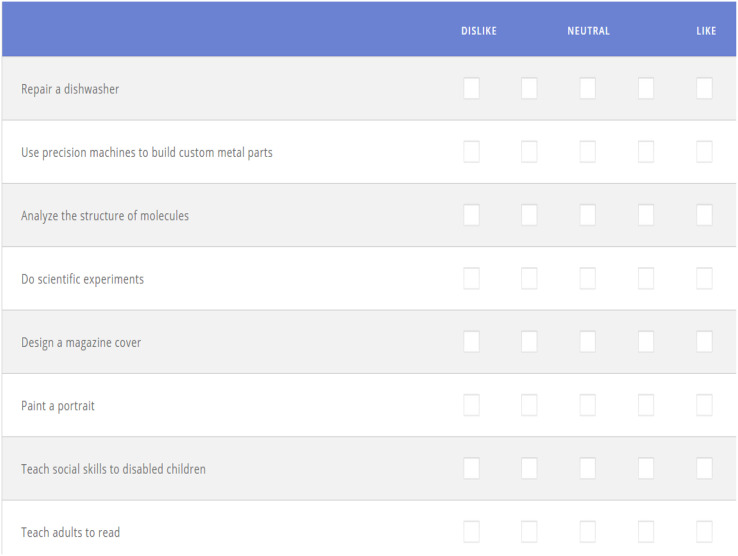
Choice survey.

Career class tests are used to dissect the classifications of participants’ careers. This test measures interest levels based on the six classes of jobs (Brown, 2002), like realistic/building, fixing, working outside, analytical/thinking, investigating, and testing. In this test, there were 20 types of affirmations to which the participants had to reply. Participants had to choose the appropriate answer based on their opinion of their career, not how they might want their job to be. Figure 4 illustrates examples of questions available in the career classification test.

### Planning test

According to Holland (1997), an individual’s ability can be exploited. Gardner (Alao Kazeem & Ibam Onwuka, 2017) put forward the idea of multiple intelligences. He stated that eight human bits of intelligence could be produced in life. According to Gardner, all these intelligence are human. They made the ninth more existential wisdom. Gardner’s multiple intelligence theory describes human knowledge in eight separate fields:

- Verbal language (VL): the capacity to understand multiple languages.
- Logical mathematics (LM): the ability to logically evaluate problems, solve math problems and examine the scientific.
- Visual and spatial (VR): the ability to visually construct a sense on article or in your mind.
- Kinesthetic (KB): able to solve a problem using body movement.
- Media (MZ): musical works specialized in performing arts and technical soundtracks.
- Interpersonal (IE): the ability to understand others’ thoughts, motives, and behaviours.
- Intrapersonal (IA): the ability to understand ourselves.
- Naturalistic (NA): knowledge of wild species that live in the world has been established.

The arranging test incorporated numerous insight tests. The hypothesis of multiple insights was created in 1983 by Dr. Howard Gardner (Vogel & Awh, 2008). It suggests that the traditionally considered understanding, based on I. Q. testing, is minimally effective. In light of everything, Dr. Gardner proposed eight particular experiences to address a broader extent of human potential in youths and adults. These experiences are:

- Linguistic insight (“word keen”);
- Logical-numerical insight (“number/thinking brilliant”);
- Spatial insight (“picture shrewd”);
- Bodily-Kinesthetic knowledge (“body keen”);
- Musical insight (“music brilliant”);
- Interpersonal insight (“individuals shrewd”);
- Intrapersonal knowledge (“self-shrewd”);
- Naturalist knowledge (“nature brilliant”).

Many pieces of information have suggested a fundamental shift in how our schools are conducted. It recommends that teachers be equipped to convey their lessons in various methods, including music, adaptive learning, hands-on activities, imaginative exercises, intelligent media, field excursions, introspection, and much more (Gardner, 1993). Speculation exists about different pieces of information significantly impacting adult learning and development. Many adults find themselves in circumstances where they cannot use the most profound insights they have ever had. For example, a compassionate person locked in an academic or mundane job might be happier in a position that allows them to wander about, such as being a sports pioneer, a woods officer, or a travel adviser. Many insights may help adults look at their life in a new way and discover opportunities they may have overlooked in their youth but can now be explored *via* courses or other activities of self-improvement.

**Figure 5:**
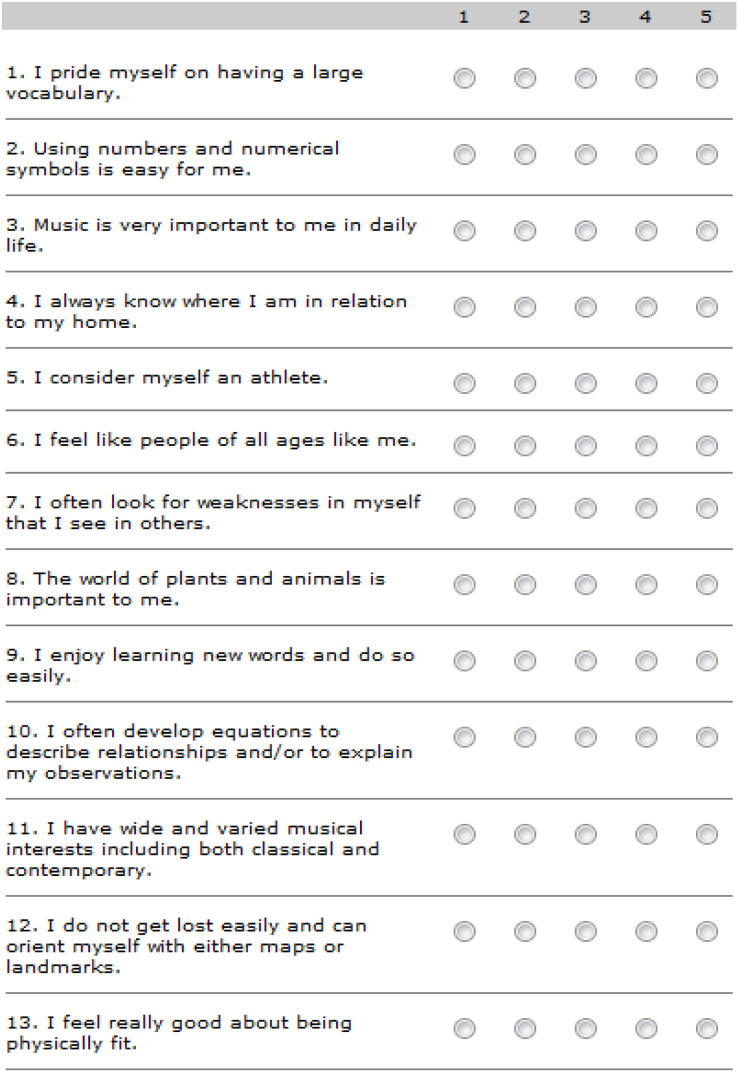
Choices survey.

Figure 5 shows examples of affirmations in the arranging test.

### Learning style

The term “learning style” refers to how each individual learns. How a student ingests, measures, fathoms, and retains knowledge defines their learning style. Individualized learning styles have gained widespread acceptance in educational theory and executive study hall methods. Individual learning styles are based on psychological, enthusiastic, and natural factors unrelated to information.

**Figure 6:**
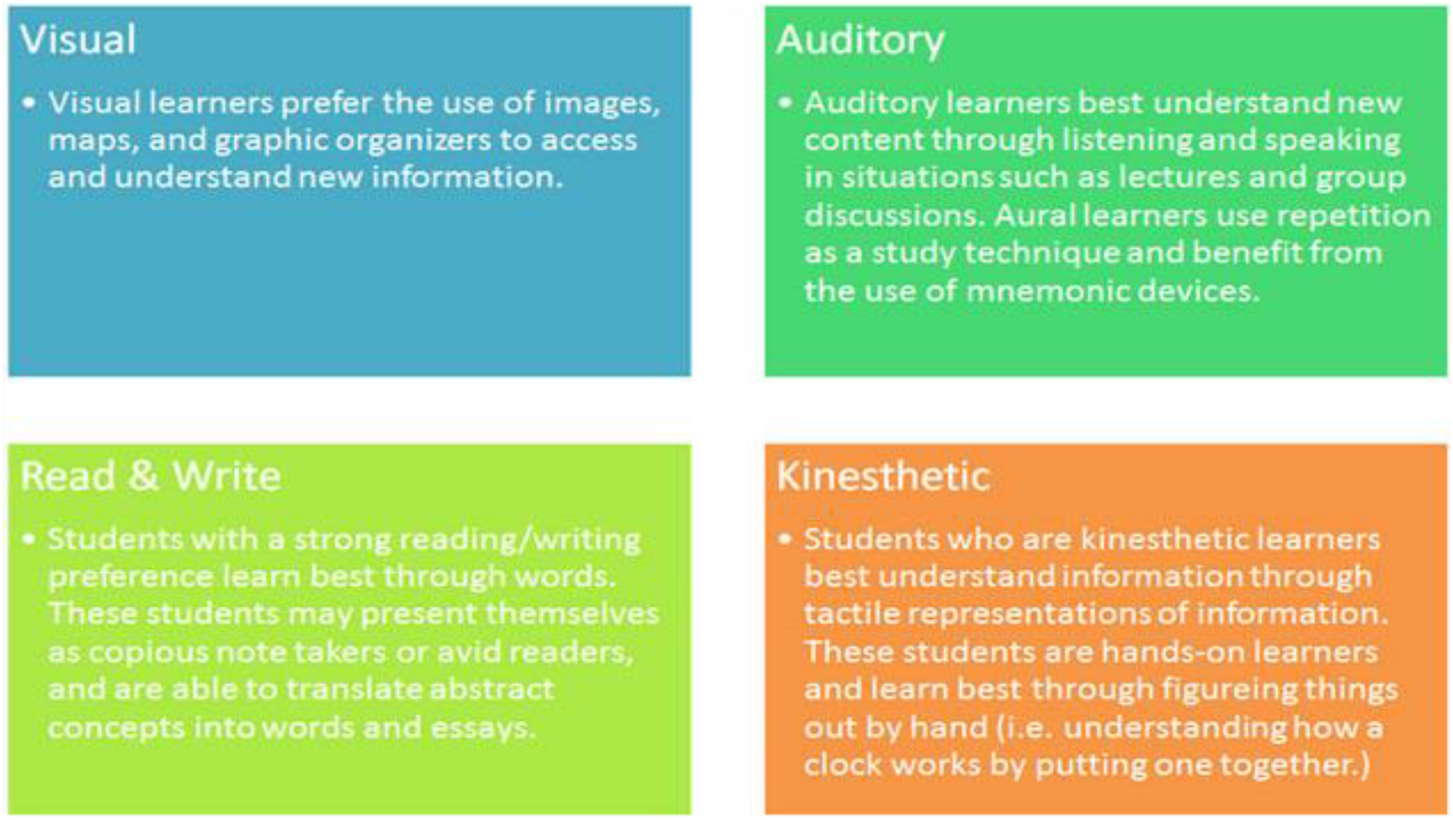
Classification students.

The VARK (visual, aural, read/write, and kinesthetic) model quickly and easily measures one’s learning style. As a result, Gardner (1993) looked into the possibility of using VARK learning styles to predict college students’ educational choices based on self-reported survey responses. The findings revealed that VARK teaching techniques might be used to match preferences for educational activities in a dependable manner. The VARK model is presented in given in Fig. 6.

The VARK model is four-dimensional. It consists of visual, auditory, read/write and kinesthetic styles (Gardner, 1993). These styles should not be thought of as separate ones, but they can be combined.

#### Methodology

The validity of the study tool was confirmed by presenting it to several experts. The experts were eligible to analyse the data that was collected and the results were calculated on the data analysis. It was further updated in its final form based on their opinion. Data reliability was checked using Cronbach’s alpha since its purpose is to examine how items (questions) are related to each other as a group. According to previous studies, the threshold level is 0.6, *i.e*., if the alpha value is greater than 0.6, then the data can be claimed as reliable (Rajaram, 2020). We obtained a Cronbach’s alpha value greater than 0.7, which justifies that the data are reliable. The correlation coefficient to check data variability was also examined. Results indicated that the correlation between each item was higher than 0.6, occurring in the range of 0.68 to 0.89 (Lehman, 2019). This shows that the inter-item correlations are significant with a *p*-value less than 0.05 and indicates sufficient data (Cronbach, 1947).

#### Usability testing

These are the steps that were followed to do usability tests:

1. Plan the test
2. Show it to experts
3. Pre-test arrangements
4. Tasks
5. Post-test
6. Results

The plan was to conduct a system usability test using actual users. These participants had not seen the system before and used it for the first time during the test.

The proposed system was tested with real-life participants who would be potential system users (*n* = 238). The details are provided in Table 1.

**Table 1:** Results.

Statement	Group	Number	Percentage
Gender	Male	179	75.21
	Female	59	24.79
College	Computer and information technology	40	16.81
	Engineering	31	13.03
	Education	24	10.08
	Science and humanities	31	13.03
	Community college	23	9.66
	Applied medical sciences	38	15.97
	Business administration	31	13.03
	Pharmacy	14	5.88
	Medicine	6	2.52
Field	Science and engineering	120	50.42
	Health	58	24.37
	Business	31	13.03
	Humanity	29	12.18
Did they successfully complete all the tasks?	Yes	238	62.47
	No	143	37.53

Three hundred eighty-one participants started the tasks. One hundred forty-three participants did not complete the tasks, so their answers were rejected. Two hundred thirty-eight questionnaires were completed. Participants’ demographics, *i.e*., their gender, college and field, were asked at the beginning of the questionnaire. They were asked whether they completed all tasks.

From two hundred thirty-eight participants, 75.21% were male respondents and 24.79% were female. There were nine colleges in total: 16.81% of participants were from Computer and Information Technology College, 15.97% were from Applied Medical sciences, 13.03% from Engineering and 13.03% from Business Administration, and the lowest percentage was for Medicine College with 2.52%. From four fields, 50.42% of participants were from Science and Engineering, 24.37% from Health, 13.03% from Business, and 12.18% were from Humanity. 62.47% of participants marked Yes to completing the tasks successfully, while 37.53% said they did not.

Participants were provided with pre-formulated test tasks to perform, and they were tested using the system based on their own requirements. In addition to that, data were collected with interviews and questionnaires after usability testing was performed.

#### Test procedure

The test was conducted online, and each participant used the system individually. Lecturers were asked to encourage students to do the tasks and answer the questionnaire.

Participants were not allowed to get help in performing tasks. Still, they could ask for any technical support or explanation to facilitate the test without intervening in their understanding or evaluation. Moderators explained the purpose and procedure of the test to the participants at the beginning.

After all, tasks were completed, participants were asked to fill out a user satisfaction questionnaire.

1. Pre-test arrangements

The following steps were conducted before the start of the test:

1. The purpose of the test was explained;
2. Procedures were explained;
3. Participants were told that the total time for the test session was 1 h;
4. They were told their rights to stop at any time without explanation;
5. They were asked whether they had any questions.

2. Performing tasks

The following steps needed to be followed to achieve the objectives of the tasks. It was ensured that all participants had the necessary equipment for the tasks. Moreover, continuous instruction checking was maintained to ensure good task completeness. Observations of the think-aloud protocol were minutely conducted.

3. Post-test tasks

The following steps were carried out once the test was successfully completed:

1. Participants were asked to fill in the post-test questionnaire;
2. Participants were thanked;
3. After the participants left, their first impressions of the test were discussed.

4. Tasks rating

Participants were asked to rate the difficulty during the test while tasks were performed with the system. The information is available in Table 2. The rating scale was as: Very Hard, Hard, Moderate, Easy and Very Easy.

**Table 2:** Degree of approval for various tasks.

No.	Phrases	F	Degree of approval						Standard deviation	Rank
		%	Very easy	Easy	Moderate	Hard	Very hard	Arithmetic mean		
1	Task 1	F	135	58	40	3	2	4.35	0.8619	2
		%	56.72%	24.37%	16.81%	1.26%	0.84%			
2	Task 2	F	75	66	38	32	27	3.55	1.3548	4
		%	31.51%	27.73%	15.97%	13.45%	11.34%			
3	Task 3	F	68	106	43	13	8	3.89	0.9902	3
		%	28.57%	44.54%	18.07%	5.46%	3.36%			
4	Task 4	F	135	78	16	7	2	4.42	0.8108	1
		%	56.72%	32.77%	6.72%	2.94%	0.84%			

Note:

User replies towards the questions for the tasks in the study.

Task 1: sign up and login

Task 2: planning test

Task 3: value test

Task 4: learning styles

Overall, Task 1 was easy for participants, with 56.72% marking it very easy and 24.37% marking it easy. Only 1.26% chose hard, and 0.84% very hard. The remaining 16.81% were unsure whether Task 1 was easy or hard. Some difficulty was faced by participants for Task 2, with 13.45% indicating that it was hard and 11.34% as very hard. Very easy was chosen by 31.51% of participants, with 27.73% choosing easy. This suggests that Task 2 was not as easy as Task 1. Task 3 was considered easier as it was easy for 44.54% of participants and very easy for 28.57%. 18.07% were not sure. This task was hard for 5.46% of participants and very hard for 3.36%. Task 4 showed the highest ease of all tasks, as 56.72% of participants marked it very easy and 32.77% marked it easy. Only 2.94% and 0.84% scored hard and very hard, respectively, while 6.72% were unsure.

The following questionnaire was given to the participants after completing all the tasks.

#### Questionnaire

The questionnaire consisted of five items on a Likert scale, ranging from strongly agree to strongly disagree, on the evaluation of the system, more especially on accessibility, user interface, information quality, satisfaction with the results, and usefulness to discover a career as per the Table 3.

**Table 3:** The questionnaire consisted of five items on a Likert scale, ranging from strongly agree to strongly disagree, on the evaluation of the system, more especially on accessibility, user interface, information quality, satisfaction with the results, and usefulness to discover a career.

No.	Phrases	F	Degree of approval					Arithmetic mean	Standard deviation	Rank
		%	Strongly agree	Agree	Neutral	Disagree	Strongly disagree			
1	This system was easy for me	F	76	109	31	15	7	3.97	0.9848	4
		%	31.93%	45.80%	13.03%	6.30%	2.94%			
2	The appearance of the system was pleasant	F	114	92	25	4	3	4.30	0.8222	1
		%	47.90%	38.66%	10.50%	1.68%	1.26%			
3	I was able to find what I was looking for	F	55	98	35	47	3	3.65	1.0793	6
		%	23.11%	41.18%	14.71%	19.75%	1.26%			
4	The information provided by the system is valuable to me	F	80	99	32	25	2	3.97	0.9845	5
		%	33.61%	41.60%	13.45%	10.50%	0.84%			
5	I was satisfied with the results	F	108	95	32	2	1	4.29	0.7603	2
		%	45.38%	39.92%	13.45%	0.84%	0.42%			
6	The system was useful to discover my career	F	110	67	21	38	2	4.03	1.1266	3
		%	46.22%	28.15%	8.82%	15.97%	0.84%			
Average		4.04								

Note:

User reply analysis for the study conducted.

*This system was easy for me*: 45.80% of participants agreed that the system was easy, with 31.93% strongly agreeing. Of the participants who did not find the system easy to use, 6.30% disagreed that it was easy to use, and 2.94% strongly disagreed. A total of 13.03% of participants answered neutrally.

*The system’s appearance was pleasant*: 47.90% and 38.66% strongly agreed and agreed, respectively, while only 1.68% and 1.26% disagreed and strongly disagreed, respectively. A total of 10.50% were neutral.

*I could find what I was looking for*: 23.11% and 41.18% of participants strongly agreed and agreed. A total of 19.75% of the respondents disagreed and 1.26% strongly disagreed. A total of 14.71% of them were neutral about this.

*The information provided by the system is valuable to me*: the information was useful for 33.61% (strongly agreed) and 41.60% (agreed) of participants, but 10.50% and 0.84% said that they disagreed and strongly disagreed, respectively. A total of 13.45% were neutral.

*You were satisfied with the results*: 45.38% strongly agreed and 39.92% agreed, respectively, that the results were satisfactory, while only 0.84% and 0.42% disagreed and strongly disagreed, respectively. With that in mind, 13.45% were neutral.

*The system helped discover my career*: 8.82% of participants were not sure that the system was useful, but 46.22% and 28.15% strongly agreed and agreed. A total of 15.97% disagreed, and only 0.84% strongly disagreed.

From the results, it can be said that most participants strongly agreed or agreed that the system was useful. It can be concluded that the questions from the questionnaire were reliable and valid for the study. Two hundred and thirty-eight participants were selected, from which we obtained information about the tasks undertaken in using the system. Results showed more male participants than females in this study, whose colleges were mostly about applied medical sciences and computer and information technology. Four tasks were asked of the participants. According to them, Tasks 1 and 4 were overall easier than Tasks 2 and 3. Looking at all the statistics, it can be seen that all tasks were relatively easy. Six questions about the system were asked of the participants after completing their studies. This section showed that most participants were optimistic about the system. Thus, it is concluded that the system was easy, pleasant, informative, satisfactory, and valuable for discovering one’s career.

#### Limitations

There are some limitations to the current study. The system is not complete. Only parts needed for research were ready, so reported programming bugs were not mentioned in this article. The programming bugs observed in the system were related to the self authentication and calculation of the results by the system. However the analysis done give the favorable results as stated in the previous sections. This study did not include two tasks, as they required special requirements.

## Conclusion and future perspectives

Choosing the correct career stream is a significant choice throughout life. Stream idea assists individuals with selecting an appropriate degree. A fulfilling future for students is essential for the human advancement. In this article, a structure to help students choose their stream in advanced education was proposed. This system asks users to pick their careers without any problem. Different difficulties in manual career guidance systems have been considered while offering this successful online career guidance system. Other streams were evaluated based on seven pre-characterized criteria. In this system, earlier instructive information, three distinct kinds of tests, learning styles, and coaching were incorporated to make the system recognize participants’ interests and limits. Viable implementation of the proposed system will help students in advanced education pick the school stream that best suits them. This system will satisfy the need to choose the right career.

## Appendix

Appendix 1. Detailed model.

**Figure 7:**
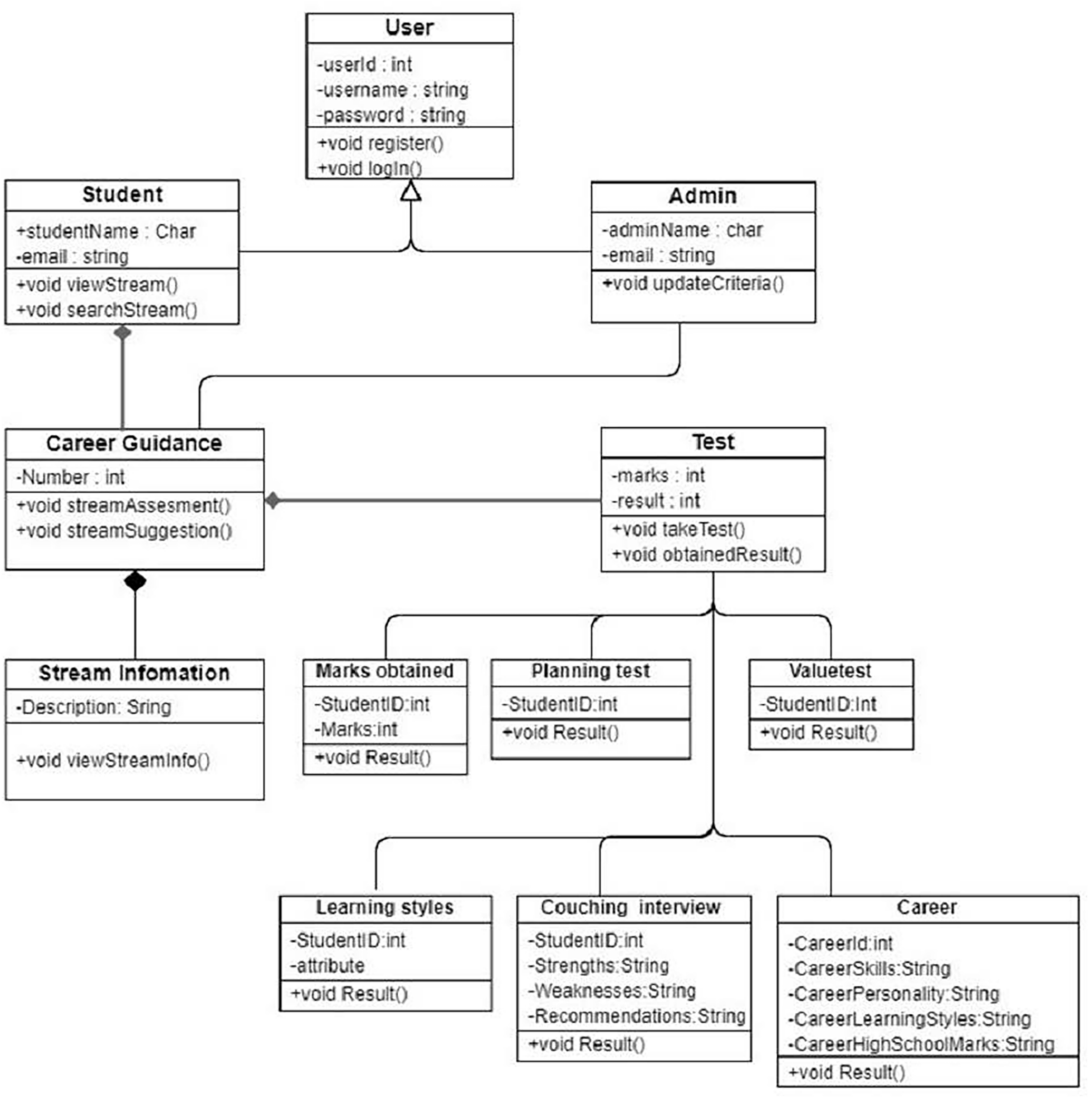
The system description for classes diagrams.

Appendix 2. Detailed model including student data.

**Figure 8:**
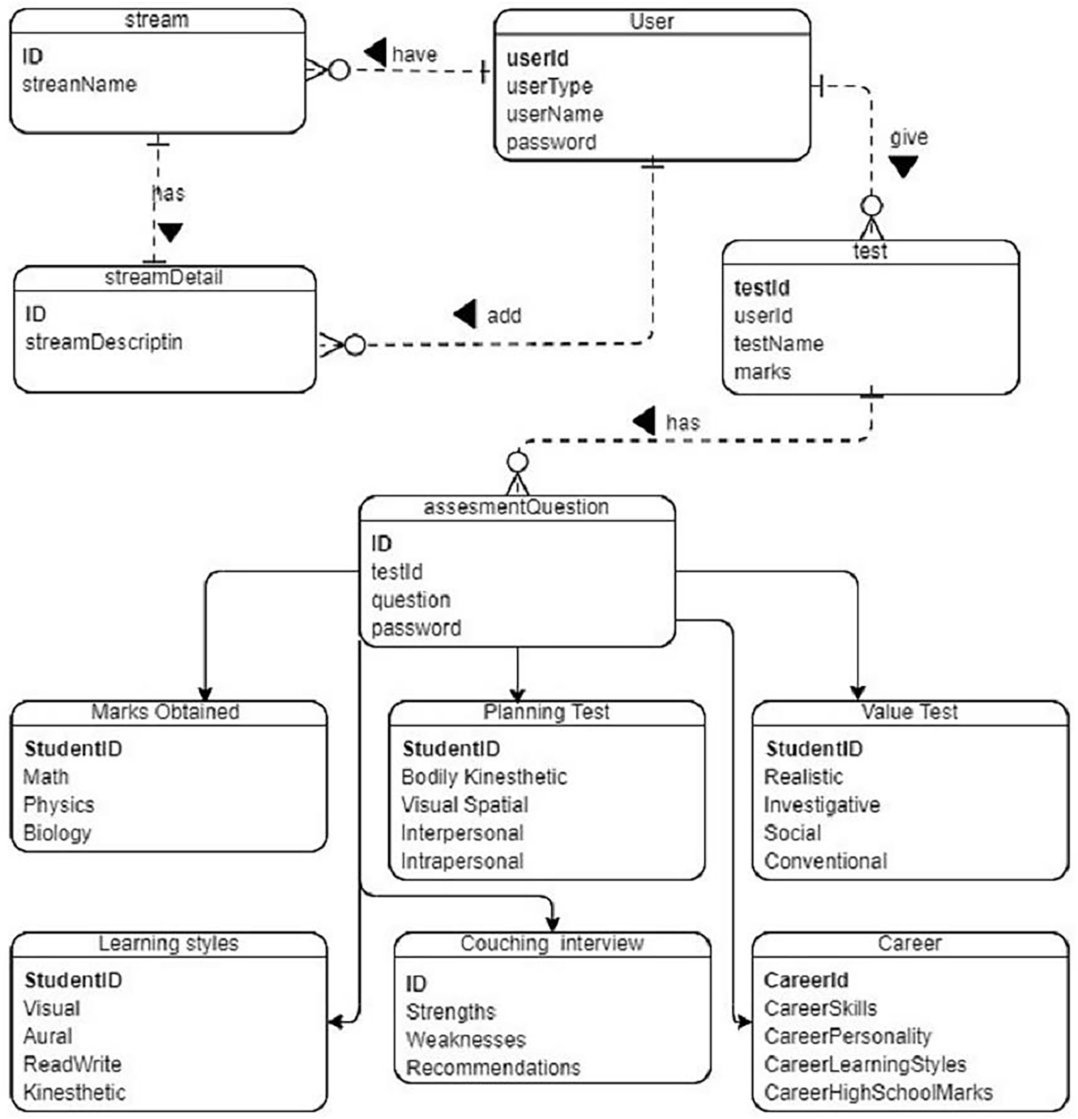
Database relationships in the system designed.

## Supplemental Information

10.7717/peerj-cs.1256/supp-1 Supplemental Information 1 Raw Dataset and Code. The code used for the analytics in this study and the results from the survey of 238 participations collected from nine different colleges in the fields of computing, engineering, education, science, applied medical, pharmacy and medicine college. The gender, learning styles and tasks responses of respondents were recorded.

10.7717/peerj-cs.1256/supp-2 Supplemental Information 2 The survey questions that were given to all the participants of the survey.

## Additional Information and Declarations

### Data Availability

The following information was supplied regarding data availability: The data is available in the Supplemental File.
