# Supplementary material for: A novel intelligent agent-based framework for appropriate stream selection from perceptive of career counseling
Source: PeerJ Comput Sci. 2023 Feb 22;9:e1256. doi: 10.7717/peerj-cs.1256 (PMC10280512; doi:10.7717/peerj-cs.1256)
Supplement: Supplemental Information 2 [file peerj-cs-09-1256-s002.pdf]

A Novel Intelligent Agent-based Framework for Effective Stream Selection: A Career Guidance Perspective – Survey Form

Demographic variables

Gender	Male<br>Female
College	Computer and information technology<br>Engineering<br>Education<br>Science and humanities<br>Community college<br>Applied medical sciences<br>Business administration<br>Pharmacy<br>Medicine
Field	Science and engineering<br>Health<br>Business<br>Humanity
Did they successfully complete all the tasks?	Yes<br>No

Tasks rating

Tasks	Very Hard	Hard	Moderate	Easy	Very Easy
Task1					
Task2					
Task3					
Task4					

Usability Questions

	Strongly agree	Agree	Neutral	Disagree	Strongly disagree
This system was easy for me					
The appearance of the system was pleasant					
I was able to find what I was looking for					
The information provided by the system is valuable to me					
I was satisfied with the results					
The system was useful to discover my career					
